# Supplementary material for: Demonstration of a beam loaded nanocoulomb-class laser wakefield accelerator
Source: Nat Commun. 2017 Sep 8;8:487. doi: 10.1038/s41467-017-00592-7 (PMC5591198; doi:10.1038/s41467-017-00592-7)
Supplement: Supplementary file 1 — Supplementary Information [file 41467_2017_592_MOESM1_ESM.pdf]

Type of file: pdf

Title of file for HTML: Supplementary Information

Description: Supplementary Figures, Supplementary Notes, and Supplementary References

----

Type of file: pdf

Title of file for HTML: Peer Review File

Supplementary Figures

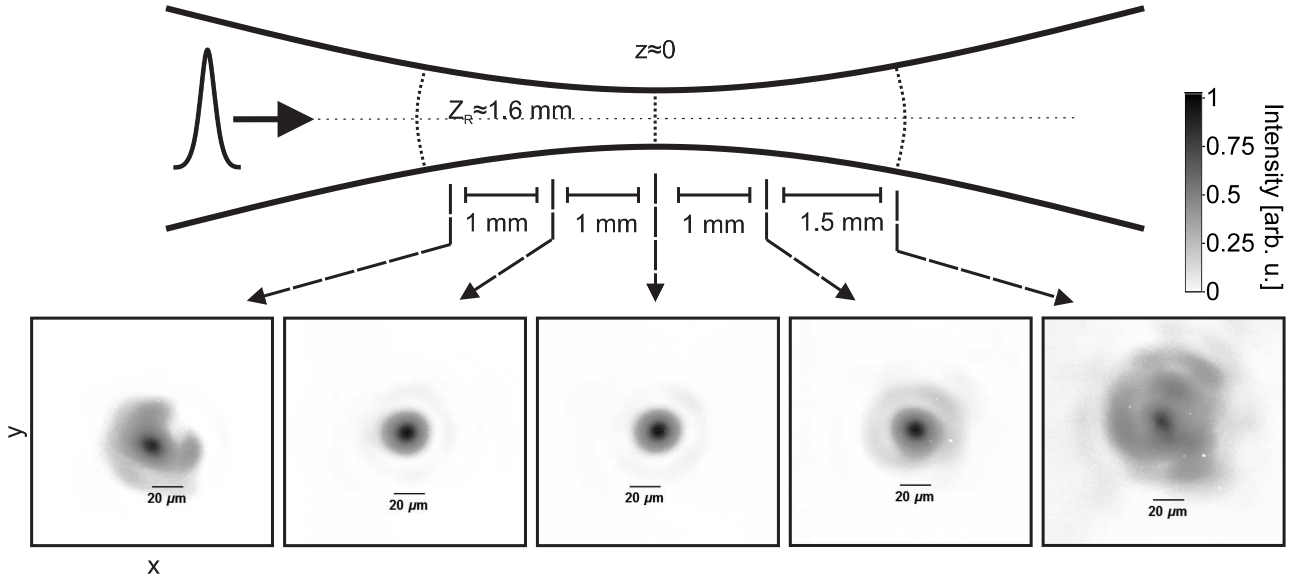

Supplementary Figure 1: Laser beam profile in vacuum at various positions around the focus. The profile was acquired with all laser amplifier stages fully pumped under experimental conditions and after phase optimization in a closed-loop using an acousto-optic programmable dispersive filter (DAZZLER-Fastlite). At the focus a spot size of 20 µm FWHM is reached. About 76% of the laser energy is within the beam waist ($1/e^2$ of intensity), corresponding to an estimated strehl-ratio of 0.9.

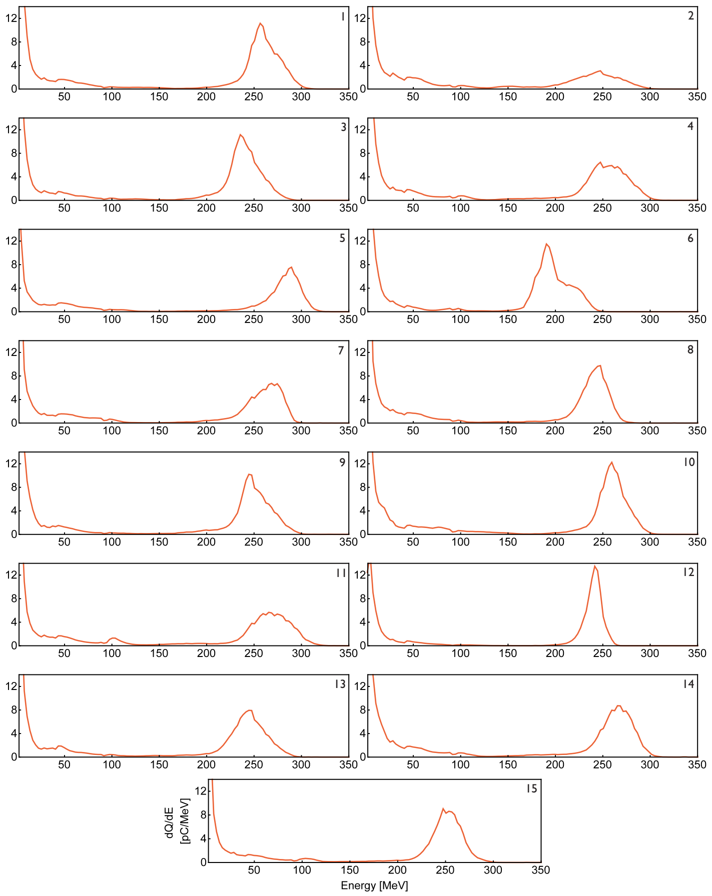

Supplementary Figure 2: Energy spectra of all 15 consecutive shots shown in figure 1(a). Axes labels are equal for all graphs and are shown only in the bottom graph. Shot numbers are shown in the top right of each graph and correspond to the shot numbers found in figure 1(a). Obtained with supersonic gas-jet with a 1.6-mm-long plasma density plateau of 3.1×10^{18} cm^{-3}, 1% nitrogen doping, 2.5 J laser energy in 30 fs FWHM duration

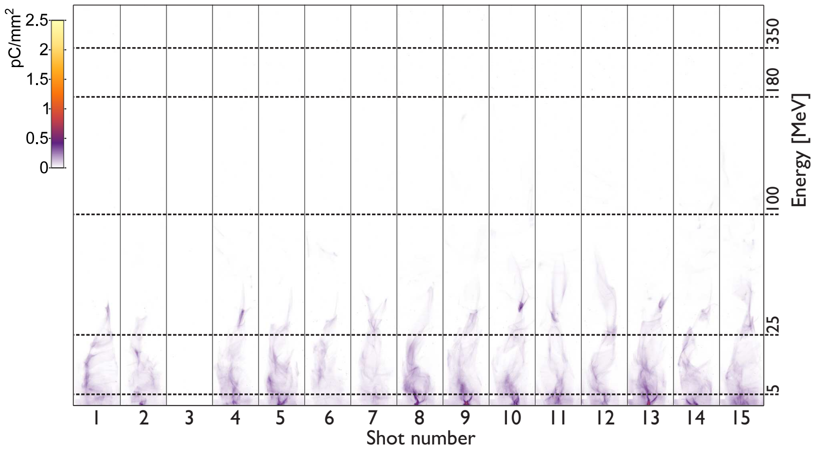

Supplementary Figure 3: Energy spectra of 15 consecutive shots shown in pure helium. No nitrogen doping is present. Obtained with a supersonic gas-jet with a 1.6-mm-long plasma density plateau of 3.1×10^{18} cm^{-3}, 3.3 J laser energy in 30 fs FWHM duration

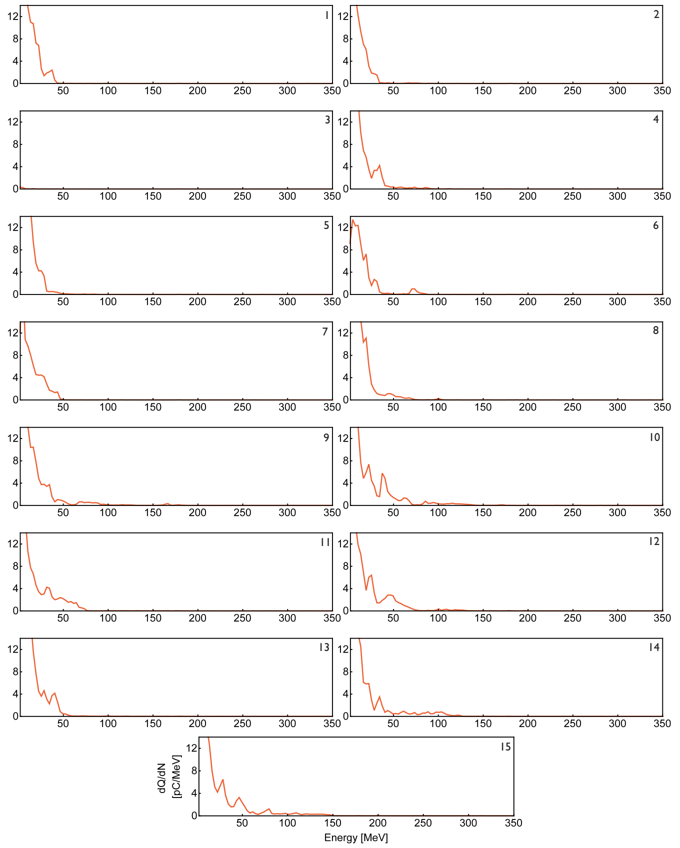

Supplementary Figure 4: Energy spectra of all 15 consecutive shots shown in supplementary figure 3. Axes labels are equal for all graphs and are shown only in the bottom graph. Shot numbers are shown in the top right of each graph and correspond to the shot numbers found in supplementary figure 3. Obtained with a supersonic gas-jet with a 1.6-mm-long plasma density plateau of 3.1×10^{18} cm^{-3}, pure helium (no nitrogen doping present, 3.3 J laser energy in 30 fs FWHM duration

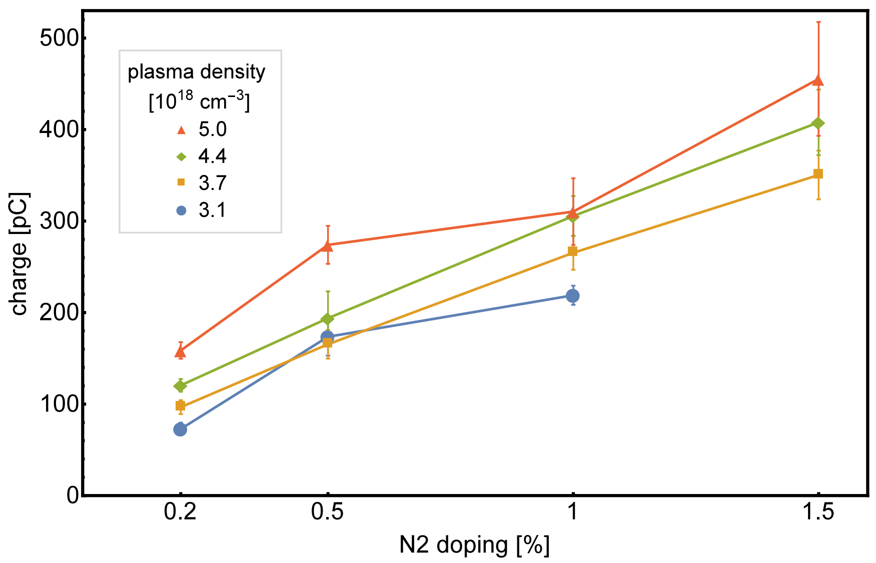

Supplementary Figure 5: Injected charge dependency. Shown is charge within the FWHM of the energy peak for different nitrogen doping concentrations and plasma densities. Data points represent the mean value from a set of shots (sample size between seven to twenty) at equal experimental parameters. Connected data points show a set of equal plasma density. The error bars represent the standard error of the mean.

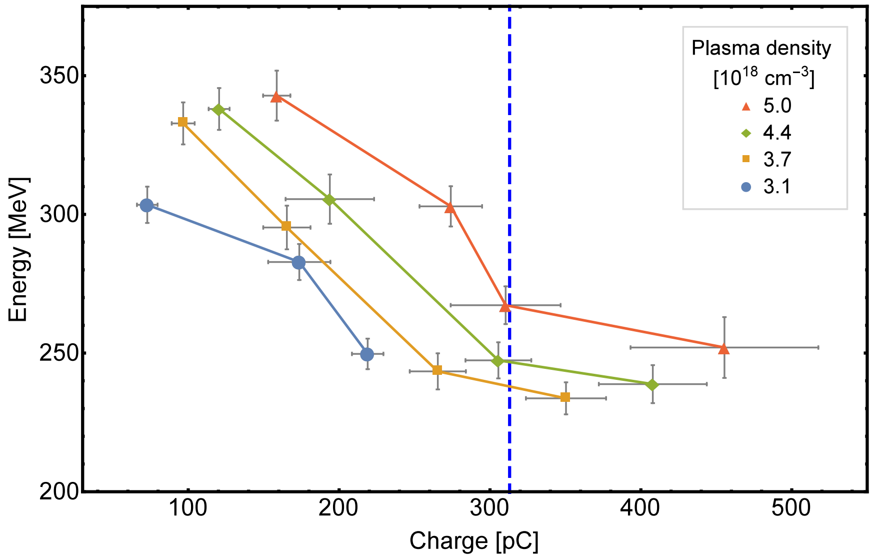

Supplementary Figure 6: Mean peak electron energy dependency. Dependency with respect to both charge within the FWHM of the energy peak as well as plasma density is shown. Data points represent the mean value from a set of shots (sample size between seven to twenty) at equal experimental parameters. Connected data points show a set of equal plasma density. The error bars represent the standard error of the mean. The estimated optimal load according to equation (1) of 313 pC is indicated by the dashed vertical line.

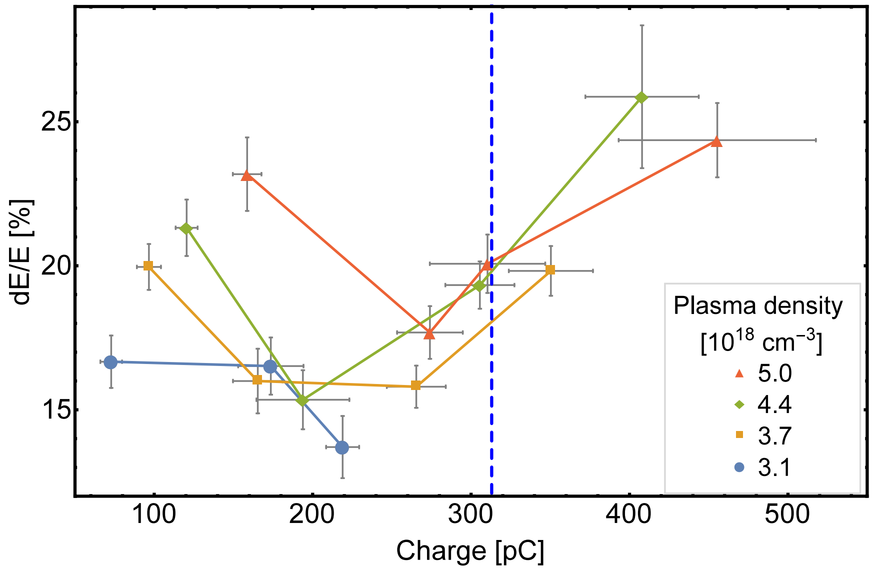

Supplementary Figure 7: Beam energy spread relative to the mean peak energy. Dependency with respect to both charge within the FWHM of the energy peak as well as plasma density is shown. Data points represent the mean value from a set of shots (sample size between seven to twenty) at equal experimental parameters. Connected data points show a set of equal plasma density. The error bars represent the standard error of the mean. The estimated optimal load according to equation (1) of 313 pC is indicated by the dashed vertical line.

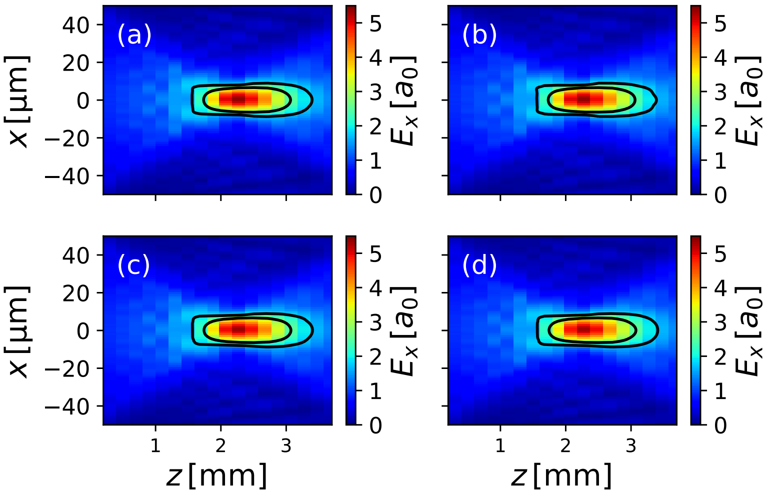

Supplementary Figure 8: Laser field strength evolution from PIConGPU simulations. Evolutions for different nitrogen doping concentrations are shown. The inner and outer region, indicated by black lines, represent the regions where the field is sufficiently high to ionize the two nitrogen K-shell electrons. Subfigure (a) corresponds to figure 4(a) in the main manuscript. All subfigures correspond to the subfigures of figure 5 in the main manuscript, with the following charges injected in the peak due to different nitrogen dopings: (a) 60 pC (b) 103 pC, (c) 142 pC (d) 168 pC.

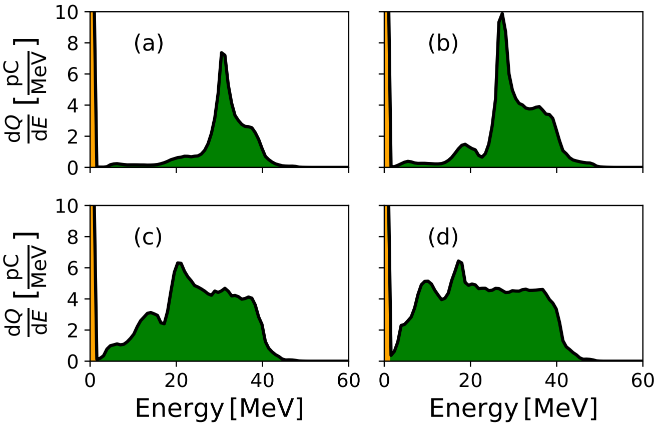

Supplementary Figure 9: Electron energy histograms from PIConGPU simulations. Histograms are assessed right after injection at z = 1.9 mm. Subfigure (a) corresponds to figure 4(a) in the main manuscript and contains a charge of 60 pC in the main peak after acceleration. All subfigures correspond to the subfigures of figure 5 in the main manuscript, with the following charges in the peak: (a) 60 pC (b) 103 pC, (c) 142 pC (d) 168 pC.

Supplementary Notes

Supplementary Note 1: Derivation of dependencies

Equation (1) from the main paper computes

$$\frac{Q_\mathrm{s}}{1\mathrm{nC}}\frac{eE_\mathrm{s}}{m_\mathrm{e}c\omega_\mathrm{p}} \simeq 0.047\sqrt{\frac{10^{16}\mathrm{cm}^{-3}}{n_\mathrm{p}}}(k_\mathrm{p}R_\mathrm{b})^4, \tag{1}$$

where c is the speed of light, Q_s is the total loaded charge, m_e and e are the mass and charge of an electron, n_p and $\omega_\mathrm{p} = \sqrt{(4\pi n_\mathrm{p} e^2)/m_\mathrm{e}}$ are the density and frequency of the plasma with the plasma wavenumber k_p. E_s is the field strength under optimal loading condition and R_b is the bubble radius. Taken that the wakefield is driven in the nonlinear bubble or blow-out regime under matched spot size conditions as described by Lu *et al.*[1] the following holds true:

$$k_\mathrm{p}R_\mathrm{b} \simeq 2\sqrt{a_0} \quad \text{and} \quad a_0 \simeq 2(P/P_\mathrm{c})^{(1/3)}. \tag{2}$$

Here $P_\mathrm{c} = 17\omega_0^2/\omega_\mathrm{p}^2[\mathrm{GW}]$ is the critical power for relativistic self-guiding and the laser normalized vector potential a_0.
In the unloaded case the maximum accelerating field gradient is given by:

$$\frac{eE_\mathrm{z,max}}{mc\omega_\mathrm{p}} = \sqrt{a_0}. \tag{3}$$

In the optimal loading condition[2], the self-field of the injected bunch has to compensate $E_\mathrm{z,max}$, i.e. $-E_\mathrm{s} = E_\mathrm{z,max}$, so that

$$\frac{eE_\mathrm{s}}{m_\mathrm{e}c\omega_\mathrm{p}} = \frac{k_\mathrm{p}R_\mathrm{B}}{2} \simeq \sqrt{a_0}. \tag{4}$$

From supplementary equations (2), (4) and $\omega_\mathrm{p} \propto \sqrt{n_\mathrm{p}}$ follows $E_\mathrm{s} \propto n_\mathrm{p}^{2/3}$.
By inserting supplementary equations (2) and (4) into supplementary equation (1) follows

$$Q_s = 1.504\frac{1}{\omega_0}\sqrt{\frac{32\cdot 10^{16}[\mathrm{cm}^{-3}]\pi e^2}{17m_\mathrm{e}}}\sqrt{P[\mathrm{GW}]}. \tag{5}$$

Taking our experimental peak power of 64 TW leads to $Q_\mathrm{s} = 313$ pC.

Supplementary Note 2: Laser spot evolution

In the STII scheme used in the experiment described in the main text, laser-plasma interaction starts before the vacuum focus is reached. This scheme requires a high quality evolution of the laser profile. Supplementary Figure 1 shows the laser profile evolution in vacuum around the laser focus. In our Particle-In-Cell simulations this laser profile was approached as accurate as possible using a Gaussian envelope. Ongoing efforts aim for more accurate future simulation studies by approaching the experimentally measured laser profile evolution more precisely using Laguerre-Gaussian modes.

Supplementary Note 3: Operation at pure helium

Supplementary Figure 3 and 4 show spectra obtained in pure helium for comparable (slight increase in laser energy) experimental parameters as for the results presented in the main article (figure 1) with nitrogen doped helium. The absence of high energy electrons in the pure helium case further illustrates that we are operating in the self-truncated ionization injection regime in the nitrogen doped case.

Supplementary Note 4: Influence of injection on the beam energy spread

The laser self-focusing and plasma dynamics are mainly influenced by the laser interaction with the background plasma. Within a dataset, plasma dynamics are kept constant and the injected charge is varied only by tuning nitrogen doping concentration (0.2%-1.5%). The injection volume is located at the front-side of the bubble, where the laser peak intensity ionizes nitrogen K-shell electrons, and then rapidly slip to the backside of the bubble, subsequently loading and/or deforming the plasma bubble. However, an influence of cavity deformation on the injection length cannot be completely excluded. Supplementary figure 8 shows that at equal plasma density and laser parameters the ionization region radius and length are independent of nitrogen doping. Although ionization happens within a large region, injection occurs only over a limited length (see main text figure 4). From our simulations we cannot extract the exact injection volume due to our resolution limit, but we can assess the beam dynamics directly after injection. Supplementary figure 9 represents the same cases presented in figure 4 & 5 of the main text and shows the electron energy distribution in the bubble evaluated right after injection (z = 1.9 mm) for different amounts of injected charge. It can be seen that injection of more charge contributes to an additional energy spread right after injection, pointing to a suspected increase of injection volume.

Comparing the energy spread right after injection and at the end of the acceleration process (figure 5 of the manuscript), we find that the contribution of energy spread from injection is small compared to the contribution of the acceleration process.
At 60 pC injected charge we find an absolute energy spread of $\sim$10 MeV right after injection, which in this weakly loaded case grows strongly to 150 MeV during the acceleration process. In contrast, at 168 pC of injected charge the accelerating field is altered further due to beam loading, leading to only a slight increase of the absolute energy spread during the acceleration process, from $\sim$35 MeV right after injection to 60 MeV after acceleration. Thus we can conclude that although the injection volume might vary depending on the injected charge, this effect is negligible compared to the effect from the acceleration process itself.

Supplementary References

[1] Lu, W. *et al.* Generating multi-GeV electron bunches using single stage laser wakefield acceleration in a 3D nonlinear regime. *Phys. Rev. ST Accel. Beams* **10**, 061301 (2007).
[2] Tzoufras, M. *et al.* Beam loading by electrons in nonlinear plasma wakes. *Phys. Plasmas* **16**, 056705 (2009).
